# ‘Real-world’ health care priority setting using explicit decision criteria: a systematic review of the literature

**DOI:** 10.1186/s12913-015-0814-3

**Published:** 2015-04-17

**Authors:** Ian Cromwell, Stuart J Peacock, Craig Mitton

**Affiliations:** Canadian Centre for Applied Research in Cancer Control, British Columbia Cancer Agency, Vancouver, Canada; Department of Cancer Control Research, British Columbia Cancer Agency, Vancouver, Canada; School of Population and Public Health, University of British Columbia, Vancouver, Canada; Centre for Clinical Epidemiology and Evaluation, University of British Columbia, Vancouver, Canada

**Keywords:** Programme budgeting and marginal analysis, Multi-criteria decision analysis, Health care priority setting, Health priorities, Health care rationing, Health care decision making, Literature review, Literature synthesis

## Abstract

**Background:**

Health care decision making requires making resource allocation decisions among programs, services, and technologies that all compete for a finite resource pool. Methods of priority setting that use explicitly defined criteria can aid health care decision makers in arriving at funding decisions in a transparent and systematic way. The purpose of this paper is to review the published literature and examine the use of criteria-based methods in ‘real-world’ health care allocation decisions.

**Methods:**

A systematic review of the published literature was conducted to find examples of ‘real-world’ priority setting exercises that used explicit criteria to guide decision-making.

**Results:**

We found thirty-three examples in the peer-reviewed and grey literature, using a variety of methods and criteria. Program effectiveness, equity, affordability, cost-effectiveness, and the number of beneficiaries emerged as the most frequently-used decision criteria. The relative importance of criteria in the ‘real-world’ trials differed from the frequency in preference elicitation exercises. Neither the decision-making method used, nor the relative economic strength of the country in which the exercise took place, appeared to have a strong effect on the type of criteria chosen.

**Conclusions:**

Health care decisions are made based on criteria related both to the health need of the population and the organizational context of the decision. Following issues related to effectiveness and affordability, ethical issues such as equity and accessibility are commonly identified as important criteria in health care resource allocation decisions.

**Electronic supplementary material:**

The online version of this article (doi:10.1186/s12913-015-0814-3) contains supplementary material, which is available to authorized users.

## Background

Health care decision making requires the balancing of the demand for programs, services, and technologies that improve human health, and the need for fiscal restraint and the reality of a finite resource pool. All health care decision making involves making choices between many attractive alternatives, and saying ‘no’ to some things that might be desirable and valuable.

Because health care resources are, in many jurisdictions, provided through public subsidy, health care decision makers have an ethical obligation to allocate those resources in a way that is fair, transparent, and accountable [1]. Further, health care decisions should be made according to the available evidence relating to a number of possible decision making criteria, including effectiveness, cost-effectiveness, equity considerations, feasibility, affordability etc. of the proposed program, service, or technology. But allocation decisions are also influenced by a number of factors other than medical and health economic evidence – pragmatic issues of organizational structure and political realities are legitimate and important components of these decisions [2-6] and must be considered as well.

Two popular proposed methods for guiding the setting of health care resource allocation priorities are Programme Budgeting and Marginal Analysis (PBMA) and Multi-Criteria Decision Analysis (MCDA). PBMA involves the listing of all relevant activities and their resource requirements, the evaluation of the effectiveness of these activities according to a set of explicit criteria, and the application of that evaluation to the available budget [7]. The PBMA process can be summarized by asking five questions [8]:

1. What resources are available in total?
2. In what ways are these resources currently spent?
3. What are the main candidates for more resources and what would be their level of effectiveness?
4. Are there any areas of care which could be provided to the same level of effectiveness but with fewer resources to fund candidates from (3) (i.e., addressing technical efficiency)?
5. Are there areas of care which, despite being effective, should receive fewer resources because a proposal from (3) is more effective (per dollar spent) (i.e., addressing allocative efficiency)?

PBMA exercises are commonly conducted by an advisory panel of expert stakeholders, and should be accompanied by an evaluation of the outputs to ensure allegiance with the priorities and needs of the organization [9].

MCDA involves the numerical quantification of the merit of competing options, according to explicit decision criteria [10]. The primary aim of MCDA is to develop models of decision maker objectives and their value trade-offs so that options under consideration can be compared with each other in a consistent and transparent manner [11]. A key principle is that decisions between different options (for example different interventions) should be consistent with stakeholders’ objectives. MCDA is transparent in that it shows that decisions are the logical implications of those objectives. In MCDA, objectives are deemed to be within the discretion of the decision-makers. That is, they are not predetermined by some underlying theory from economics or ethics (such as utilitarianism).

MCDA then typically consists of two overarching stages [10,11]. First, problem structuring involves generating a set of alternatives and a set of criteria against which the alternatives are to be evaluated and compared. In order to structure the problem, the first questions to ask are ‘what priority setting objectives do decision-makers wish to pursue?’ And, ‘what locally relevant criteria do decision-makers use when deciding between alternative interventions?’ Objectives are the principles that determine priority setting policies (e.g. improving population health) whereas criteria are the standards that alternative interventions are judged by (e.g. health outcomes from different treatments). Second, model building entails constructing some form of model which represents decision-makers’ objectives and their value judgements. There are then two key considerations to be addressed in this type of model [10,11]: the methods used to describe decision-makers preferences and elicit importance weights for decision-making criteria; and, the type of aggregation model used to combine criteria scores (see Peacock 2009 for more detail).

Similar to PBMA, MCDA is conducted with the input of decision makers and relevant stakeholders in the ultimate resource allocation decision [10]. In addition, MCDA can be employed within the broader PBMA approach as the mechanism for benefit measurement to inform allocation or re-allocation recommendations. As such, these approaches in our view are best viewed as complementary.

In a time when organizations are adopting explicit criteria-based decision methods and deciding which method is the best fit for their organization [12,13], it is important to examine the criteria that have been used in previous decisions. While reviews of the priority-setting literature have been conducted [2,14], these studies have focused on hypothetical exercises and stated preferences rather than cases where decision-making bodies have had to make decisions under the actual constraints of budgetary and political realities.

The purpose of this paper is to summarize the available literature on health care decision-making where explicit criteria-based methods like PBMA or MCDA were used (i.e., a set list of factors were weighed against each other according to some underlying framework), in order to examine the criteria used by decision makers in ‘real world’, rather than hypothetical, settings. The added value of this paper is that our review, based on significant experience in this field over the past 12 years, focused on ‘real-world’ priority setting using explicit decision criteria. These examples may be interpreted as a better predictor of future health care priority setting than decisions made in the abstract.

## Methods

A systematic review of the literature was conducted, based on English language search terms used in a previously published review of this literature [15]. Relevant subject headings were abstracted from the articles included in that review: “Decision Making, organizational”; “Health Care rationing”; “Health Priorities”; “Budgets”; and “Community Health Planning”.

To broaden the scope of our search to ensure inclusion of exercises using popular decision-making methods, the terms “Program (me) Budget”, “Marginal Analysis”, and “Multi-Criteria Decision Analysis” as well as their respective acronyms (“PBMA”, “MCDA”) were also added to the search terms.

The relevant search terms were used to search the MEDLINE, ECONLit, and PAIS databases. The search terms were also entered into the Google™ search engine to investigate the presence of ‘grey literature’ (i.e., non peer-reviewed publications). Retrieval was limited to documents published between January 1^st^, 2000 and July 31^st^, 2013, to reflect the relevant time period since the previous literature review.

Articles were identified as potentially eligible based on a review of their abstracts. Potentially eligible items underwent closer examination based on exclusion criteria. Items were excluded if they did not meet the following description: a description of a priority setting exercise in which a funding decision was made based on a set of explicitly-defined criteria. Articles published in languages other than English were not included in this review.

**Figure 1:**
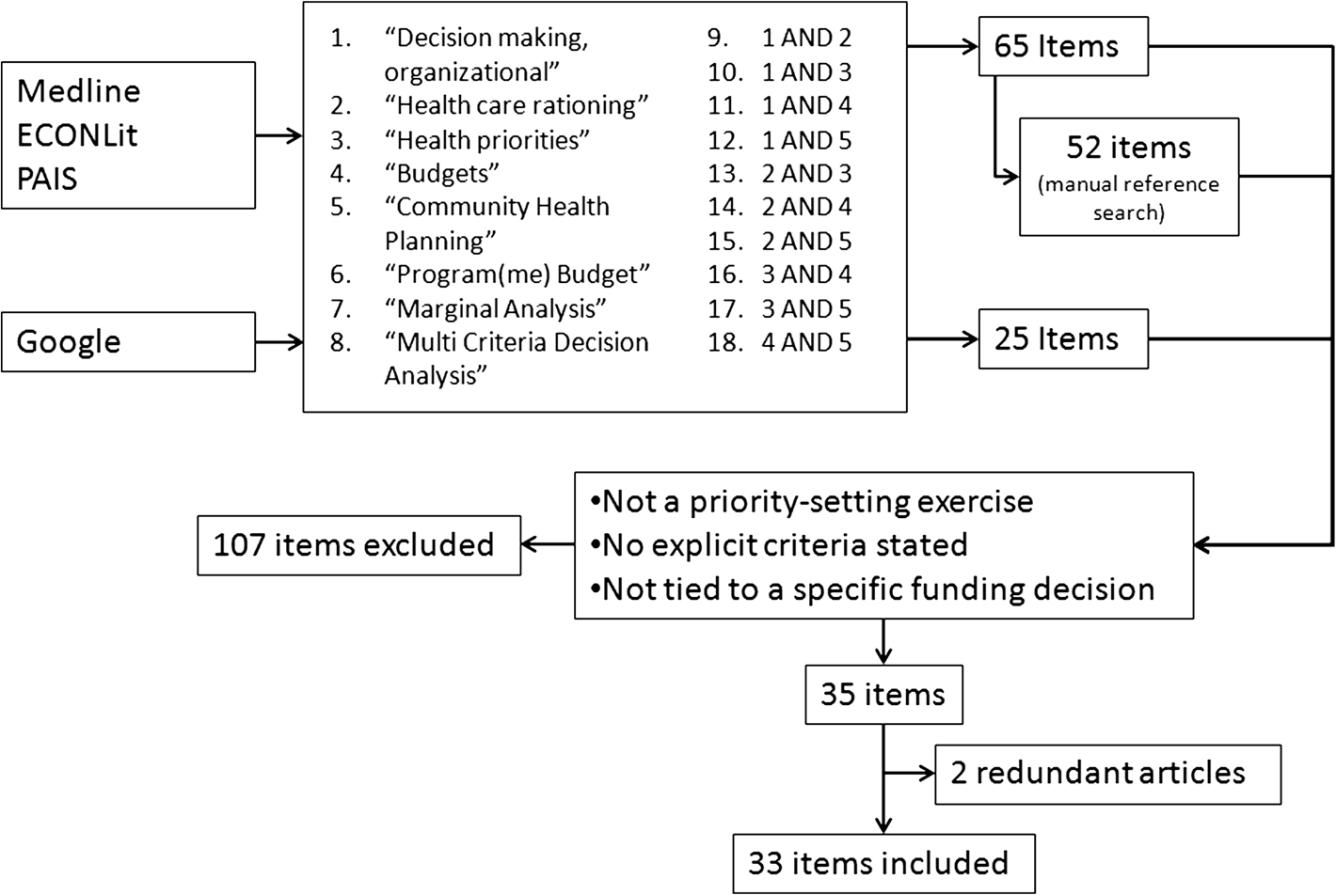
Search strategy and results.

The reference lists of all items (i.e., excluded and included) were manually searched for potentially-eligible items. The reference-identified items underwent the same scrutiny and application of exclusion criteria. The search strategy is diagrammed in Figure 1. One reviewer (IC) conducted the review and data abstraction from all studies. Two other reviewers (SJP and CM) verified the accuracy of the search process and the application of the exclusion criteria. Disagreements about eligibility were resolved through consensus. Because studies included in this exercise were descriptive rather than quantitative, and because of the nature of the research question, no formal assessment of the quality or bias of included items was undertaken. Because this research did not involve human participants, no Research Ethics approval was sought or gained.

## Results

A total of sixty-five articles were identified as potentially eligible from the indexed databases. An additional fifty-two were identified from the manual reference search and 25 from web search, for a total of 142 potentially eligible items. Manual search from the grey literature did not yield any additional unique items. After applying the exclusion criteria, thirty-five articles were identified as eligible. Two peer-reviewed items were duplicates of items from the grey literature and were deemed redundant, bringing the total number of included items to thirty-three.

### Study characteristics

Characteristics of the included studies, including the setting, method of decision-making used, and the result of the exercise (e.g., a change in policy, a list of priorities, etc.), are presented in Table 1. Most exercises were conducted in North American or European or European-descended (“Western”) countries, primarily Canada (10) and the United Kingdom (7). There was equal representation of studies using an MCDA framework (15) as using PBMA (15). Some studies did not explicitly state the decision-making method, or used a synonymous term (e.g., “Decision Science”), and were classified as PBMA or MCDA based on the characteristics of the method used (e.g., generic vs. algorithmic methods of criteria weighting and decision ranking, inclusion of budgetary information, etc.).

**Table 1:** **Summary of included items**

**Study**	**Country**	**Setting**	**Weighting method**	**Method**	**Type of decision**
[22]	Australia	Hospital	Mixture of several different methods (ratio, rating scale)	PBMA	Increased resource allocation for highly-ranked programs
[23]	Nepal	National level	Discrete Choice Experiment	MCDA	Ranking of 34 possible interventions
[24]	UK	Primary Care Trust	Allocation of points method	PBMA	Prioritizing 4 programs for diabetes care
[25]	New Zealand	Public Health System	No weights described	PBMA	5 investments, 5 disinvestments
[26]	Norway	Norwegian Health Ministry	Discrete Choice Experiment	MCDA	Ranking of 21 different alternatives among 5 health domains
[27]	Canada	Health Authority	Weights, method not described	PBMA	18 investments, 13 disinvestments, $4.5 m reallocation
[18]	Canada	Health Authority	40 points could be allocated to any of 40 items	PBMA	$16 m reallocated, $1 m released through service reduction
[28]	Canada	Not specified	Weights, method not clear	MCDA	Creation of priorities list
[29]	New Zealand	Health Authority	No weights described	PBMA	Summary of decisions
[30]	Canada	Municipal District	No weights described	PBMA	Program alternatives prioritized
[31]	USA	Health Authority	Percentages (allocation? Ratio?)	MCDA^a^	Ranking of 47 programs funded by the region
[32]	UK	2 Primary Care Trusts	Allocation of points method	PBMA	66 proposals approved that met criteria out of 134 submitted
[33]	Ghana	National level	Discrete Choice Experiment	MCDA	Ranking of 11 health programs
[34]	Canada	Provincial level	Discrete Choice Experiment	MCDA	Development of decision tool
[35]	UK	Primary Care Trust	Mix of ratio (for main criteria) and points allocation (for sub-criteria)	PBMA	£3.37 m disinvested, £2 m used for defecit reduction
[36]	Taiwan	National Health Insurance	Grey incidence mathematical expression	MCDA	Access to care optimization
[37]	Korea	Hospital	Goal programming multicriteria decision modelling	MCDA	Staffing and other logistic optimization for hospital resource allocation to meet goals
[38]	Tanzania	National Ministry of Health	No weights described	MCDA	Prioritization of 9 programs
[39]	UK	Department of (Public) Health	Discrete Choice Experiment	MCDA	Ranking of 14 different preventative health measures
[40]	South Africa	Department of Health	Rating Scale	MCDA	Evaluation of LBC as cervical cancer screening tool
[41]	Canada	Health Authority	40 points could be allocated to any of 40 items	PBMA	$40 m in resources released, used for defecit and reinvestment
[42]	Canada	Health Authority	Allocation of points method	MCDA	9 alternative programs ranked
[43]	Canada	Health Authority	Allocation of points method	PBMA	44 disinvestments, $4.9 million in cost reduction
[44]	Canada	University faculty of medicine	Allocation of points method	PBMA	55 disinvestments, $2.7 million in cost reduction
[45]	UK	Health Authority	No weights described	MCDA	Construction of optimization model; mapping of disinvestments
[46]	Canada	Surgical Department in Hospital	No weights described	MCDA	Evaluation of 53 health technologies
[47]	Canada	Surgical Services in Health Region	No weights described	MCDA	Development of decision tool
[48]	UK	Primary Care Trust	Allocation of points method	PBMA	Ranking of 7 programs with PBMA, then with ad hoc approach
[49]	Canada	Health Authority	No weights described	PBMA	Additional funding of $200,000
[50]	UK	Primary Care Trust	Allocation of points method	MCDA	Ranking of 4 program alternatives
[51]	UK	Primary Care Trust	Allocation of points method	MCDA	Ranking of 6 different alternatives
[52]	Thailand	National level	Discrete Choice Experiment	MCDA	Ranking of 40 HIV/AIDS interventions
[53]	Thailand	National level	No weights described	MCDA	Ranking of 17 possible services for inclusion in national insurance scheme

a – This paper describes its methodology as “decision science”, but the methodology is very similar to MCDA, as was therefore classified that way.

The most common outcome of MCDA exercises was a ranked list of alternatives, rather than an explicit funding decision of the type that was more common among exercises that used PBMA. Allocation of points, whereby an agreed-upon number of points can be assigned to different categories, was a common method of assigning weights to decision criteria. Many MCDA exercises used Discrete Choice experiments to elicit criteria weights. A number of studies did not describe any weighting method, and may have simply considered all criteria equally important.

### Choice of decision-making criteria

The decision-making criteria used in each included item were identified and extracted. Where possible, criteria with identical/similar definitions across different studies were collapsed into a single criterion (by IC, decisions reviewed by SJP and CM), to make direct comparison between exercises more possible. A total of seventy-two unique criteria were identified among the included items. These criteria are listed in Additional file 1: Appendix A. The most common criteria were:

- The effectiveness of the program (21 items)
- Budgetary impact/Affordability (16 items)
- Reducing inequalities between groups/”Equity” (14 items)
- Number of people likely to benefit from program/intervention (13 items)
- Ability to access the program/intervention (13 items)
- Cost-effectiveness or other health economics evidence (12 items)
- Quality of the available evidence (10 items)

It is important to note that, because of variations in the way in which criteria were described, it may be possible that some criteria are ‘redundant’, insofar as some may simply be more specific iterations of others. For example, the criteria of “poverty reduction” and “age/risk of target group” could both be collapsed into the “Equity” criterion. A conservative approach was taken to combining criteria in this way, preferring to list criteria individually in cases where there was ambiguity about whether or not terms were truly synonymous. Similarly, the classification of a given criterion into a domain was prone to subjectivity – criteria were not always well-described in the text of the exercise. We again used a conservative approach for this classification process, and resolved ambiguities through consensus.

It should also be noted that many studies listed broad criteria that contained a number of sub-criteria within them (e.g., one study listed an “effectiveness” criterion that included “number of patients”, “individual benefit”, “magnitude of benefit”, “duration of benefit”, “personal networks”, “collective benefits”, “population impact”, and “social capital” as included sub-criteria). In cases where criteria weights were not provided for each sub-criterion, the broad criteria were preferred.

### Domains of decision-making criteria

Criteria used on priority setting exercises were classified into ten descriptive ‘domains’, using a classification system described by Tanios and colleagues [14]:

- Intervention outcomes and Benefits
- Type of Health Service
- Disease Impact (burden)
- Therapeutic Context
- Economic Impact
- Environmental Impact of the Intervention
- Quality/Uncertainty of Evidence
- Implementation Complexity
- Priorities (fairness)
- Overall Context

**Figure 2:**
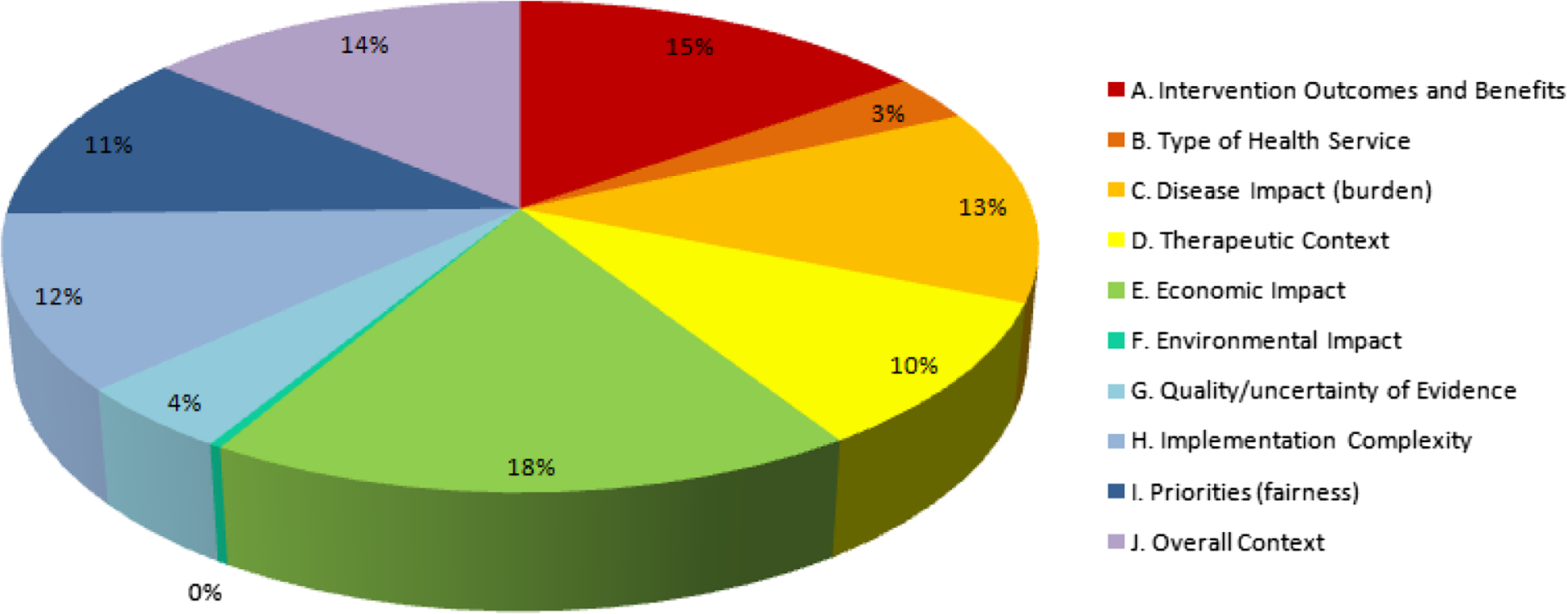
Decision criteria by domain.

A full list of all criteria included in each domain is provided in Additional file 1: Appendix A. A proportional breakdown of each domain is provided in Figure 2. Economic Impact and Intervention Outcomes/Benefits were the two most frequently-cited domains, followed by Overall Context, Disease Impact (burden), and Priorities (fairness). Only two of the included items included a Miscellaneous category (i.e., a category labeled “miscellaneous” in the report/manuscript itself).

### Domain by type of exercise

**Figure 3:**
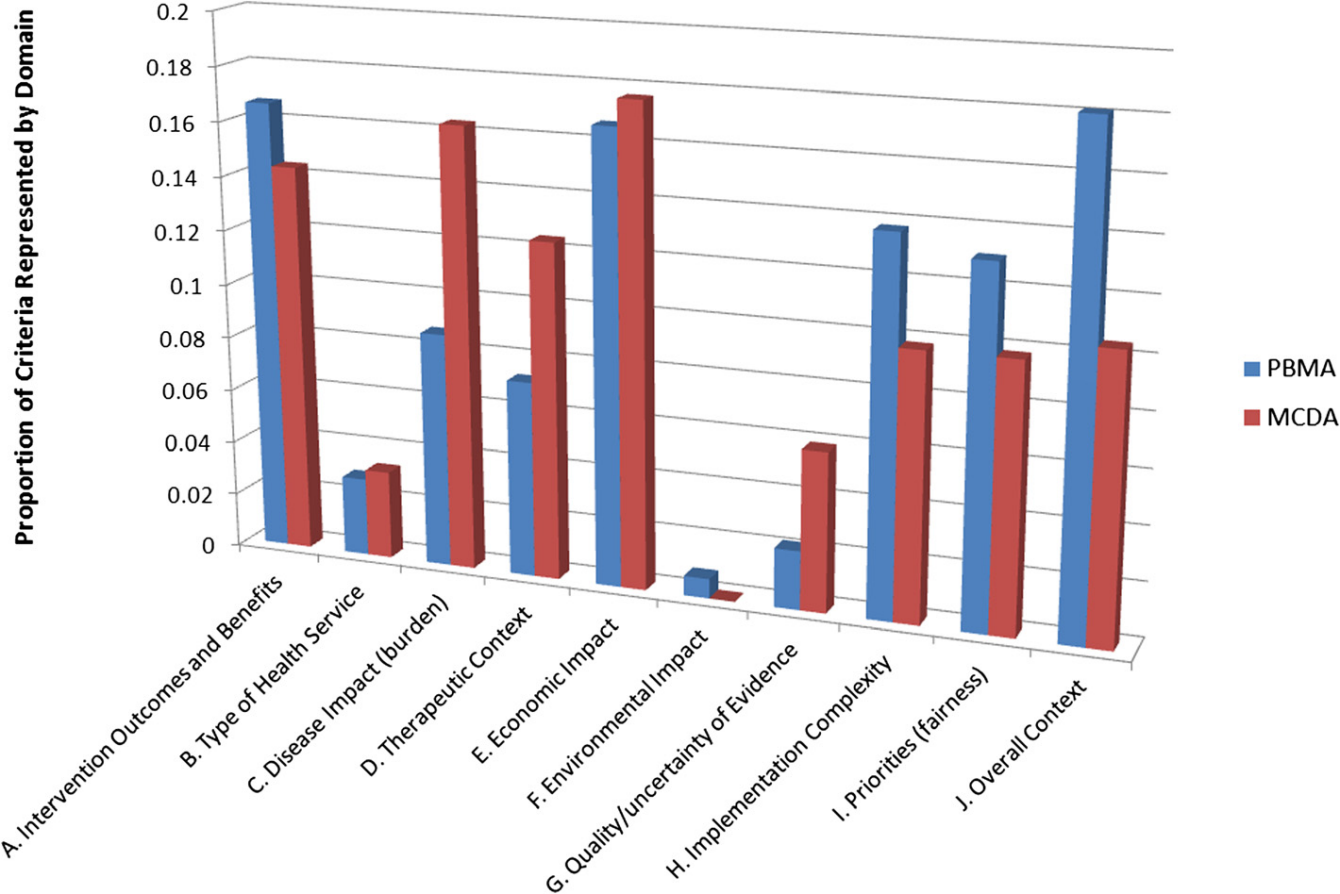
Criteria domain frequency by priority-setting method (PBMA vs. MCDA).

It is possible that the types of criteria used may differ due to the differences in the setting and methodology between PBMA and MCDA exercises. The frequency of criteria use within each domain was calculated for each type of approach. Results are presented in Figure 3. Overall, the types of criteria used were similar across the exercise types. Differences between the proportion of domain in each exercise were not significant (two-tailed t-test; α = 0.05).

### Domain by country of origin

**Figure 4:**
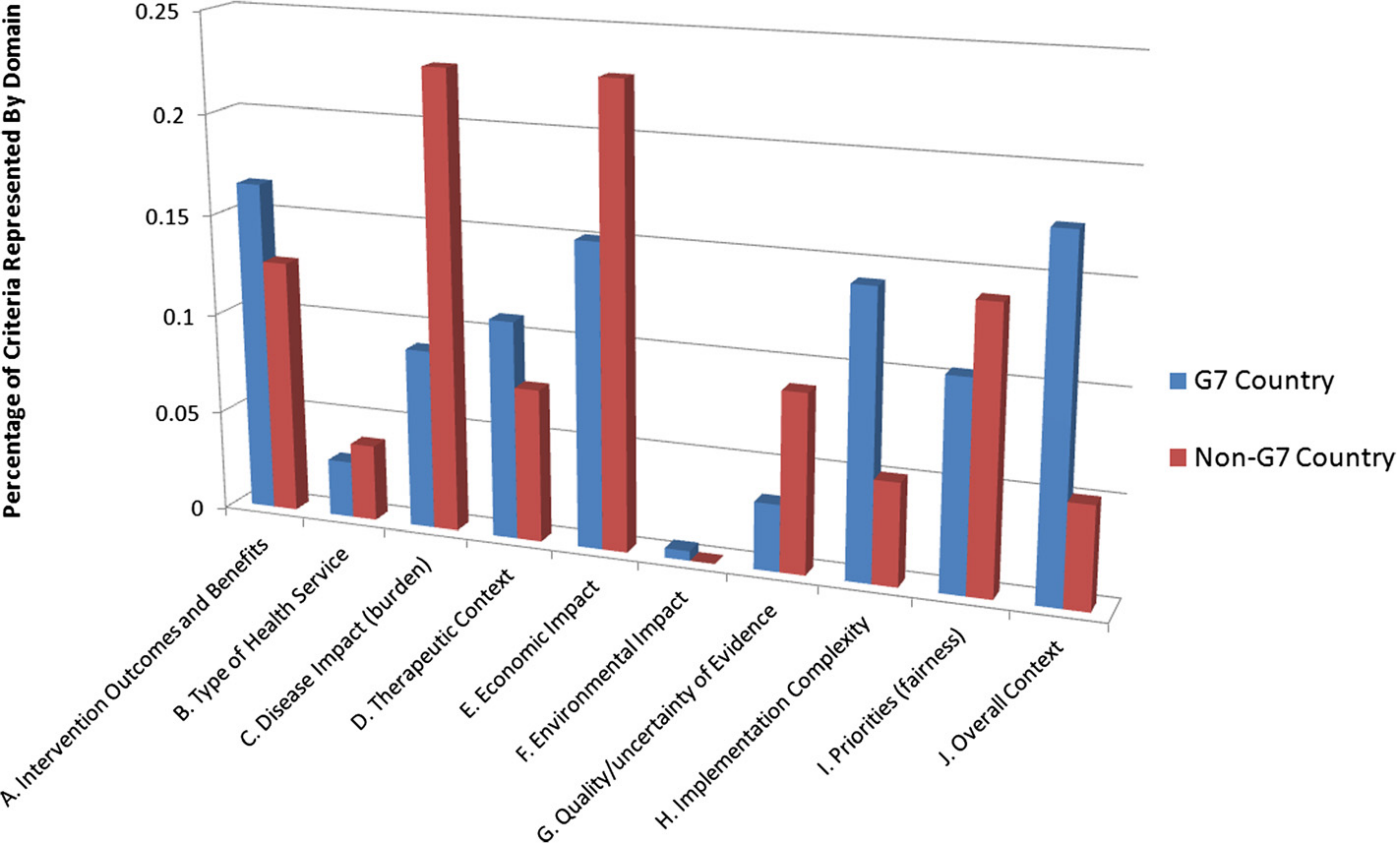
Criteria domain frequency by size of national economy (G7 vs. non-G7).

It is similarly possible that the size of the national economy affects the types of criteria used in health care decisions. Exercises in our study were grouped based on membership in the G7 group of countries (Canada, France, Germany, Italy, Japan, the United Kingdom, and the United States). The frequency of criteria use within each domain was calculated for exercises conducted in G7 and non-G7 countries. Once again, the frequency of criteria was largely similar between the two groups. Exercises performed in non-G7 countries were more likely to consider criteria in the Disease Impact (Burden) domain than those in G7 countries (two-tailed t test, p = 0.002); conversely, exercises in G7 countries were more likely to consider criteria in the Overall Context domain (p = 0.006) – other differences were not statistically significant. Results are presented in Figure 4.

## Discussion

Our search yielded 33 distinct ‘real-world’ priority setting exercises conducted using explicit decision-making criteria. Decisions were made largely along common categories of criteria that included the likely impact of a program/intervention, the ability of a program/intervention to address inequalities, and the political/organizational realities of the entities that are making funding decisions.

### Summary of main findings

Our review found that while health-specific decision making criteria are of primary importance in priority setting exercises, criteria relating to organizational and political considerations (i.e., criteria that fall primarily into the “Implementation Complexity” and “Overall Context” domains) are also important elements in decision making. Health care delivery must be guided by the available biomedical evidence, but as it is still a human endeavour, political realities must be considered and weighed. Decision makers should be forthcoming about the need to balance the goal of improving human health with the pragmatic constraints all health systems are under.

It is worth noting that fewer than half of the items included in this review explicitly included cost-effectiveness evidence in their decision-making process. While it is certainly true that costs and outcomes are considered in other criteria (i.e., Affordability, Effectiveness of program), incremental cost-effectiveness is not a component of these analyses. Health economic evidence is uniquely suited to provide valuable information when making decisions about scarce resources, but was not explicitly considered in the majority of the identified exercises. This finding is in line with a growing body of research suggesting that while decision-makers would like to use health economic evidence, they may not be able to do so [16,17]. Health economists and policy makers must continue to work together to determine the best way of making health economic findings more policy-relevant and usable.

It is valuable to note that, after “effectiveness of the program” and “affordability”, issues related to equity and fairness were foremost in the decision-making process. This suggests that, at least where decision criteria are made explicit, decision makers are concerned with addressing systemic inequalities and providing health care on a ‘by-need’ basis, underlining the important role of ethics in health care decision making [4,18].

We found that, broadly speaking, the studies in this review fell equally into two categories: MCDA exercises and PBMA exercises. It is important to note that a number of studies did not explicitly categorize themselves as either PBMA or MCDA, for example in one case using the term “Decision Science” to describe the methodology. In these cases, we classified all studies in this review as either PBMA or MCDA based on the methods described in the publication. It is conceivable, therefore, that other reviewers may classify them differently. Our discussion of the contrast between “MCDA exercises” and “PBMA exercises” should be read with this caveat in mind.

The primary outcome of the MCDA items included in this review was the prioritization of alternatives. It is not clear from the manuscripts themselves whether the priorities identified through the MCDA process became official policy, rather than simply a set of recommendations. By way of contrast, the PBMA studies often refer explicitly to investment/disinvestment actions taken as a result of the exercise. It may be valuable to policy-makers to know, when deciding what priority setting approach they prefer, how the approach translates into actual policy decisions.

Given that the resource constraints in developing economies are greater than those of countries with larger budgets, the use of criteria-guided priority setting is of particular importance in countries with developing economies [19,20]. MCDA was used in a number of countries with developing economies, whereas PBMA was used exclusively in countries with developed economies (especially Canada and the UK). The preference for MCDA appears to be due in part to the effort of investigators associated with Dr. Rob Baltussen, who is listed as an author on four of the seven relevant MCDA exercises. In parallel, application of PBMA has tended to be associated with a handful of applied researchers working in the UK, Australia and Canada. While the socio-geographic disparity seen in our review is likely to be a simple reflection of this authorship ‘clustering’ effect, it may be worthwhile to investigate whether there are barriers or relevant factors to the use of other decision-making techniques in these countries; whether there is a true difference in the suitability of PBMA or MCDA in particular political/financial resource environments.

We looked for differences in the type of criteria used in PBMA vs. MCDA exercises, as well as in G7 countries vs. the rest of the world. Care should be taken, however, in extrapolating from these results. The small number of available studies and the lack of a consistent definition of criteria mean that such comparisons are inherently difficult to make. Researchers may wish to investigate the extent to which decision-makers in wealthy countries face different pressures when allocating health resources than those faced by decision-makers in less wealthy countries. Our findings suggest that both PBMA and MCDA can incorporate the criteria that are most important to the local context, and that the decision-making method should be chosen independently of the chosen criteria, based on the feasibility and applicability of a given method to that context.

Regardless of the method of priority setting used, the use of explicit criteria is valuable for all levels of health care decision making. Because no health care system can fund all possible alternatives, decision makers have an ethical obligation to act as good stewards of finite health care resources, and should be accountable to the communities they serve.

### Limitations

Our search criteria were based on a previously-published review of PBMA exercises. As a result, it is possible that the terms are biased toward one decision-making method at the expense of others. We intentionally used MeSH terms and included an additional term that specifies MCDA in order to counteract any potential bias toward PBMA. We also used manual searches of the reference section of all papers included in the review, in order to ensure that as wide a ‘net’ was used in the search. It is worth noting that while the majority of the items included in this review were found using generic search terms (e.g., “Decision Making”, “Health Priorities”, etc.), we did supplement our search to use PBMA and MCDA-specific terms as well. It is possible, therefore, that our review is biased toward these methods. Regardless, we believe that this exercise represents a fair encapsulation of the status of the literature.

We chose to classify our criteria according to a previously-published conceptual framework [14]; however, our classification was subject to our own interpretation – criteria were not listed in consistent language, requiring us to make our own decisions. It is possible that our interpretations were biased, and that other raters would classify a given criterion differently. It is additionally possible that an interested reader would classify or combine the listed criteria differently (e.g., “efficiency” and “appropriate use of resources” may be seen as identical criteria, though this review counts them separately). The conservative approach we used – preferring to list criteria verbatim to reduce rater bias – leaves a great deal of subjectivity up to the reader. Accordingly, we have listed the criteria used in this analysis, as well as the way in which the criteria were assigned to domains, in Additional file 1: Appendix A.

The conceptual framework we used also makes these evaluations vulnerable to the bias of the raters. An alternative framework has been described by Tromp and Baltussen [21] that classifies criteria according to two broad categories – ‘health system goals’ and ‘health system building blocks’ – as well as a number of subcategories. There is a great deal of overlap between the Tanios et al. framework and the one proposed by Tromp and Baltussen, and there is a level of arbitrariness in choosing one rather than the other. Perhaps importantly, the Tromp and Baltussen framework specifically does not consider “Quality of Evidence” as an independent criterion, rather treating it as a component that underlies all criteria. Given that some type of evidence quality criterion was present thirteen times in the studies in this review, we feel that the Tanios framework accurately encapsulates the terms that health care decision makers have used to describe their work.

It is worth noting that health care decisions are often made on an ad hoc basis rather than using a specific framework like MCDA or PBMA – our review includes all published studies, which likely overrepresents the frequency with which these two approaches are used. It is unlikely that our search comprises an ‘exhaustive’ review of all criteria-based priority setting exercises in health care. As many entities within the UK’s National Health Service (NHS) have adopted PBMA as part of its decision-making process, more exercises are likely either in progress or completed without publicly-available documentation. Similarly, successes of MCDA as a priority setting method in developing countries will likely yield further use of the process in future decision-making. While not a comprehensive review of all such exercises, this review does provide insight into the types of criteria that decision-making bodies consider important when allocating scarce resources.

### Further discussion

The authors of this review are reliable advocates of PBMA as a priority setting exercise, and are named as authors on several of the studies included in this review. One advantage of using PBMA is that budgetary analysis is an intrinsic component of the priority setting process, which allows for the important process of disinvestment – the reduction of funding to programs that do not deliver acceptable value for money. As health systems and regions in various parts of the world face pressure to control, or in some cases reduce the size of their budgets, disinvestment becomes an increasingly important component of health care priority setting. As described in Table 1, several studies were able to find ways to disinvest as a method of reallocating scarce funds – all of these studies used PBMA.

A review conducted by Guindo et al. [2] explored the criteria used in resource allocation decision-making; however, the Guindo et al. review included focus groups and other activities not explicitly tied to a particular resource allocation decision, rather than those decisions made in a ‘real-world’ setting. As in our review, the criteria of equity, effectiveness, organizational requirements, and availability of cost-effectiveness literature were commonly cited as important. An important difference between the two studies, however, is that “stakeholder interests & pressures” was a highly influential criterion in the Guindo review, and not in ours. This may suggest that, though they identify it as important, decision-makers are less likely to include stakeholder input in actual decision-making exercises tied to explicit funding decisions. Of course, the overlap of papers was not great and thus there could also be other reasons for this difference including the setting and/ or individuals involved in the given work. Further investigation should be conducted into the use of stakeholder input into decision-making processes, to see how, and at what level, such preferences are incorporated into health care decision making.

This review contributes to the scientific literature in two important ways: first, it includes much of the ‘grey’ literature that is not present in previously-published reviews of this field. Because a great deal of health care decision-making is done outside the context of peer-reviewed journals, it is critical for researchers and decision-makers alike to be able to draw from as broad a set of examples as possible when trying to decide what sort of criteria are relevant to their particular context of interest. Secondly, by focusing on health care resource allocation decisions that have been made, as opposed to hypothetical exercises, our review reflects a set of decision-making criteria that is more ‘pragmatic’ than the more ‘aspirational’ criteria that may be generated in the abstract.

## Conclusions

This review points to the criteria that are most important to health care decision makers in actual policy-setting environments, and builds on previous reviews that included hypothetical decision criteria. It is important to recognize that each priority-setting environment has its own unique challenges, and the criteria used will reflect this heterogeneity. However, this review does suggest some convergence in those criteria that are most frequently used in a ‘real-world’ setting.

## Additional file

Additional file 1: Appendix A. Decision Criteria.

## Abbreviations

MCDA: Multi-Criteria Decision Analysis
PBMA: Programme Budgeting and Marginal Analysis
